# Large animal models for the study of tendinopathy

**DOI:** 10.3389/fcell.2022.1031638

**Published:** 2022-10-25

**Authors:** Guorong Zhang, Xuyan Zhou, Shuang Hu, Ye Jin, Zhidong Qiu

**Affiliations:** ^1^ School of Clinical Medicine, Changchun University of Chinese Medicine, Changchun, China; ^2^ School of Pharmacy, Changchun University of Chinese Medicine, Changchun, China

**Keywords:** large animal model, tendinopathy, rotator cuff injury, patellar tendon injury, Achilles tendon injury, flexor tendon injury

## Abstract

Tendinopathy has a high incidence in athletes and the aging population. It can cause pain and movement disorders, and is one of the most difficult problems in orthopedics. Animal models of tendinopathy provide potentially efficient and effective means to develop understanding of human tendinopathy and its underlying pathological mechanisms and treatments. The selection of preclinical models is essential to ensure the successful translation of effective and innovative treatments into clinical practice. Large animals can be used in both micro- and macro-level research owing to their similarity to humans in size, structure, and function. This article reviews the application of large animal models in tendinopathy regarding injuries to four tendons: rotator cuff, patellar ligament, Achilles tendon, and flexor tendon. The advantages and disadvantages of studying tendinopathy with large animal models are summarized. It is hoped that, with further development of animal models of tendinopathy, new strategies for the prevention and treatment of tendinopathy in humans will be developed.

## Introduction

**FIGURE 1:**
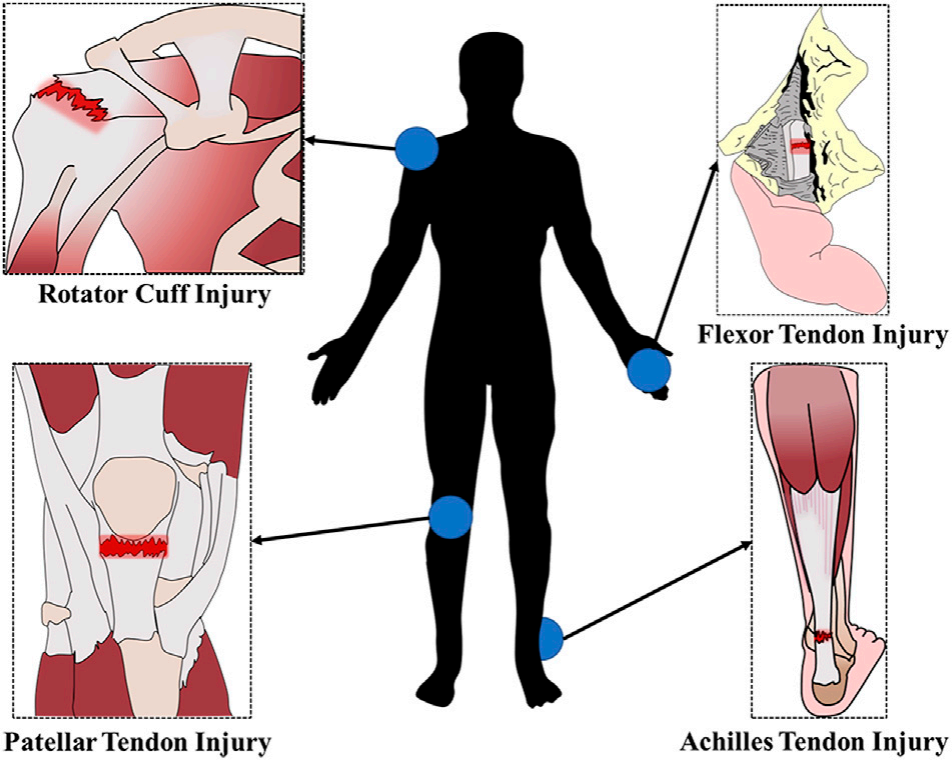
Schematic diagram of the sites of tendon injuries that frequently occur in humans.

Tendons are dense connective tissues that connect muscle to bone and transmit the forces created by muscles to the bones, which causes movement. Trauma, strenuous exercise, or overuse can lead to acute tendon rupture or chronic degenerative disease, resulting in pain, movement disorders and other clinical symptoms (Dean et al., 2017). Tendon injuries often occur in areas with frequent movements and high stress. The frequent sites of tendon injuries in humans are demonstrated the schematic diagram in Figure 1. Tendon injury is very common and can be debilitating, but tendon repair remains a clinical challenge for orthopedic medicine.

Tendon biology is poorly understood compared to other components of the musculoskeletal system (Gaut and Duprez, 2016). Development of effective therapeutics is hindered by the lack of fundamental guiding data on the biology of tendon development, signal transduction, mechanotransduction, and the basic mechanisms underlying tendon pathogenesis and healing (Andarawis-Puri et al., 2015). The ability to perform invasive assays in animal models provides researchers with powerful opportunities to improve understanding of many aspects of tendinopathy (Warden, 2007). The efficacy and safety of stem cells, growth factors, drugs, tissue-engineered tendons, and other therapeutic methods must be validated in animal models before using them in clinical trials. Therefore, to study the pathogenesis and treatment of tendinopathy, animal models are indispensable (Habets et al., 2018; Longo et al., 2018).

The pathology and pathophysiology of tendinopathy in humans is currently poorly understood, meaning that the validation of animal models is difficult. Two common and established features associated with tendinopathy in humans are histopathological changes and mechanical weakening of the tendon (Warden, 2007). The prominent histological and molecular features of tendinopathy include increased immune cells and inflammatory mediators, enhanced cellular apoptosis, dysregulated extracellular matrix homeostasis, disorganization of collagen fibers, an increase in the microvasculature, and sensory nerve innervation (Millar et al., 2021). The most reasonable way to generate tendinopathy in animal models is to introduce known and potential causative factors. At present, the most used methods of modeling tendinopathy include mechanical stimulation, chemical induction, and surgical operation.

The principal animals used in the study of tendinopathy include rats, mice, chickens, rabbits, sheep, horses, dogs, pigs, and so on. The research objectives of tendinopathy are stratified by two main concerns (Liu et al., 2021). One is at the micro level, such as the biological characteristics of tendon-bone healing, the potential signaling pathway and the genetic mechanism of the disease, *etc.*; verified small animal models can usually meet the needs of such research. The second is at the macro level, such as biomechanical modeling, optimization of surgical techniques, evaluation of new instruments or devices, *etc.* This type of research requires animal models to be highly similar to humans in structure and function, so large animal models, especially non-human primate models, are usually more suitable. In most cases, these two goals are not separate; both micro and macro levels need to be considered. Large animals have more prospects for application because they can be used for both microscopic and macroscopic research.

This article reviews and analyzes the large animal models used in the study of tendinopathy in recent years with respect to four aspects: rotator cuff injury, patellar ligament injury, Achilles tendon injury and flexor tendon injury. The article aims to provide reference for further research.

### Rotator cuff injury model

**FIGURE 2:**
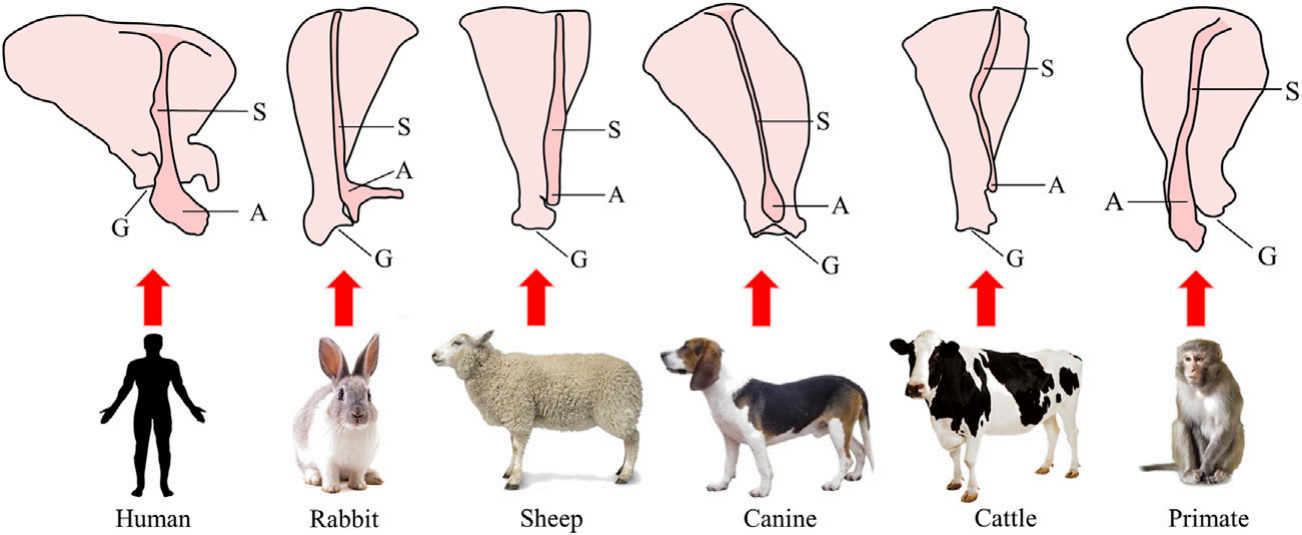
Schematic diagram of the structure of the scapula in humans and different animals. S, scapular spine; A, acromion; G, glenoid cavity.

Rotator cuff injury is the most common shoulder injury. After injury, the tissue often presents with irreversible muscle atrophy, stiffness and fatty infiltration (Derwin et al., 2007). The ideal animal model of rotator cuff injury should have a similar anatomical structure and function to humans to be able to simulate the local microenvironment and histological changes after tendon injury. Previous studies have evaluated the application of various experimental animals in rotator cuff injury modeling (Takase et al., 2017; Lebaschi et al., 2018). But, in fact, except for humans, most animals rely on limbs for support. Even if there is occasional standing behavior supported on double hind limbs for a short time, the double forelimbs still have more weight-bearing functions than humans. Thus, the anatomy of the shoulder is different between humans and most other animals. The shoulder structure of nonhuman primates is the closest anatomical and physiological analog to that of humans. A schematic diagram of the scapula structure of human and different animals is shown in Figure 2. Large animal models of rotator cuff injury are mainly rabbits, sheep, dogs and cattle, which are suitable for studying the mechanisms of muscle degeneration, stent repair technology and improved surgical methods (Bisson et al., 2008; Liu et al., 2018; Smith et al., 2018; Roßbach et al., 2020).

Rabbits are one of the commonly used animal models in orthopedic studies. Hyman et al. presented a detailed architectural and physiological analysis of chronic tear and repair compared with age-matched control rabbit supraspinatus muscles (Hyman et al., 2021). Previous studies have used rabbit supraspinatus tendon or infraspinatus tendon tear models to analyze the tendon-bone healing effects of platelet-rich plasma and ozone therapy (Gurger et al., 2021), microfracture apertures (Sun et al., 2020), decellularized tendon sheets (Liu et al., 2018), preservation of native enthesis (Su et al., 2018) and nano-calcium silicate mineralized fish scale scaffolds (Han et al., 2023). Lee et al. studied the histological and biomechanical changes in a rabbit model of chronic rotator cuff tears repaired by human dermal fibroblasts (Lee et al., 2021). Yildiz et al. used a rabbit irreparable rotator cuff tear model to compare the healing of two types of upper joint capsule reconstruction grafts (Yildiz et al., 2019). Xu et al. explored the in vivo biomechanical and histological processes of the rerouting biceps tendon to treat chronic irreparable rotator cuff tears in a rabbit model (Xu et al., 2022). In addition, Grumet et al. proposed that the rabbit subscapularis muscle model could be used for the study of rotator cuff lesions. They found that the rabbit glenohumeral joint had a bone channel formed by the supraglenoid tubercle, coracoid process and infraglenoid tubercle. The rabbit subscapularis muscle passes through this bone channel and to the lesser tubercle of the humerus, similar to the human supraspinatus tendon that passes under the acromion to the greater tubercle of the humerus. More importantly, the structure traversed within this bone tunnel is the tendon part of the subscapularis muscle. Another advantage of this model is the presence of fatty infiltration of the muscle after the tendon is severed (Grumet et al., 2009). Vargas-Vila et al. studied the progression of muscle loss and fat accumulation in a rabbit model of rotator cuff tear (Vargas-Vila et al., 2022). Wang et al. studied that adipose stem cell-derived exosomes decreased fatty infiltration and enhanced rotator cuff healing in a rabbit model of chronic tears (Wang et al., 2020).

Sheep can be used as a practical, large animal model. This model has been gradually used in various orthopedic studies, including studies on rotator cuff repair, due to its easy availability, ease of rearing, and relatively low cost. The large size of the infraspinatus tendon in sheep makes them particularly suitable for *in vitro* biomechanical studies (Camenzind et al., 2016; Guo et al., 2016). In addition, the sheep model has also been used to investigate the effect on rotator cuff repair of engineered tissue grafting (Novakova et al., 2018), perforated anchors, or collagen scaffolds loaded with tenocytes (Roßbach et al., 2020). Coleman et al. successfully constructed a chronic rotator cuff injury repair model by wrapping the broken end of the ovine infraspinatus tendon with a membrane (Coleman et al., 2003). Sener et al. used two different fixation methods to investigate the biomechanical and histological characteristics of patellar tendon-bone autografting and free flexor-tendon autografting in reconstruction of an infraspinatus defect in sheep (Sener et al., 2004). Romeo et al. evaluated the mechanical, structural, and histologic quality of rotator cuff repairs augmented with an interposition electrospun nanofiber scaffold in an acute sheep model (Romeo et al., 2022). Luan et al. found that muscle fatty infiltration and fibrosis would also occur after repair of acute rotator cuff injury in sheep (Luan et al., 2015). In a sheep rotator cuff model, tenotomy predominantly induces fatty infiltration, and denervation induces mostly muscle atrophy (Wieser et al., 2021). Some scholars have further revealed the possible mechanisms of muscle atrophy and degeneration after rotator cuff injury through sheep models, which is expected to provide a new target for clinical treatment (Gerber et al., 2017; Ruoss et al., 2018a; Ruoss et al., 2018b).

The canine rotator cuff injury model has been used to study a variety of rotator cuff repair materials or surgical methods (Adams et al., 2006; Derwin et al., 2007; Roller et al., 2018; Smith et al., 2018). Adams et al. used a full-thickness tear model of the Canis infraspinatus tendon to evaluate the effect of using human acellular dermal matrix grafting to enhance rotator cuff repair (Adams et al., 2006). Smith et al. compared the augmentation of partial rotator cuff tears of biologic scaffolds in a canine model (Smith et al., 2020). Derwin et al. evaluated the applicability of the canine rotator cuff acute full-thickness injury repair model; their results demonstrated that the repair strength immediately after surgery depended on the suture type and method, although all repaired tendons had retears shortly after surgery (Derwin et al., 2007). Derwin et al. evaluated the extent to which augmentation of acute repair of rotator cuff tendons with a newly designed poly-L-lactide repair device would improve functional and biomechanical outcomes in a canine model (Derwin et al., 2009). In addition, the canine model can replicate muscle atrophy and fatty infiltration following rotator cuff injury in humans. Safran et al. developed a canine rotator cuff chronic injury model to explore the dynamic performance, muscle volume, and fat infiltration of infraspinatus muscles over time (Safran et al., 2005). The canine rotator cuff injury repair model can also tolerate various postoperative rehabilitation programs, such as plaster fixation, suspension fixation, and treadmill exercises (Lebaschi et al., 2016). Ji et al. tested a non-weight-bearing (NWB) canine model for rotator cuff repair research. In this model, the extensor muscles of the elbow and wrist were denervated by cutting the radial nerve proximal to the innervation region of the triceps brachii, to prevent weight bearing and muscle contraction of the affected limb after surgery (Ji et al., 2015). Liu et al. reported a novel biomaterial with engineered tendon-fibrocartilage-bone composite and bone marrow-derived mesenchymal stem cell sheet; the construct was tested for augmentation of rotator cuff repair using a canine NWB model (Liu et al., 2019).

The rotator cuff injury model of cattle is used in biomechanical research. Previous studies have investigated the biomechanical differences between single-row and double-row rotator cuff repairs (Mahar et al., 2007), the initial fixation strength of different suture methods under cyclic loading (Anderl et al., 2012), the influence of suture materials on the biomechanics of the suture–tendon interface (Bisson et al., 2008), and the pull out strength of different stitches (Bisson and Manohar, 2010). Park et al. evaluated the interface pressure of the rotator cuff tendon to the greater tuberosity for different rotator cuff repair techniques. Simulated rotator cuff tears over a 1 x 2 cm infraspinatus insertion footprint were created in 25 bovine shoulders. A transosseous tunnel simple suture technique, suture anchor simple technique, and suture anchor mattress technique were used for repair. Their results showed that the transosseous tunnel rotator cuff repair technique created significantly more contact and greater overall pressure distribution over a defined footprint when compared with suture anchor techniques (Park et al., 2005). The main advantage of the bovine rotator cuff injury model is that there is little difference in rotator cuff size and quality of tissue between different individuals, which helps to ensure the consistency of the replication model (Liu et al., 2021).

From the perspective of translational medicine, nonhuman primates are undoubtedly the most ideal species for shoulder joint research, as they are the most similar to humans in anatomical structure and physiology. Plate et al. studied age-related degenerative functional, radiographic, and histological changes of the shoulder in nonhuman primates (Plate et al., 2013). Sonnabend et al. stated that baboons make the best animal model for rotator cuff repair research based on their shoulders’ similarity to the human shoulder joint (Sonnabend et al., 2010). Xu et al. used the African green monkey rotator cuff injury model to evaluate the repair effect and immune response of a non-cross-linked porcine dermal extracellular matrix graft; the results of this study demonstrated that the graft integrated well with the host tendon tissue and did not cause significant inflammatory reactions (Xu et al., 2012). However, the high cost of primate rearing, complexity of management, and difficulties in ethical approval limit its experimental application (Warden, 2007).

### Patellar tendon injury model

The patellar tendon is located on the surface of the knee joint, connecting the patella and the tibia of the lower leg. It is one of the knee extensor devices. Due to the constant movement of the human knee joint, the patellar tendon is under periodic load for a long time, and the patellar tendon has become a common and frequent site of tendinopathy (Hast et al., 2014). In general, the patellar tendon is relatively large and its anatomical position is superficial and easy to access (Longo et al., 2011). The animal model of this part is convenient for biomechanical testing and research because the dual osseous attachment of the patellar tendon can be pulled without direct injury to the tendon material. However, the patellar tendon is wide and thin, which makes it difficult to inject drugs into. It requires delicate local operations, which require higher technical requirements for operators. Large animal models used in the study of tendinopathy include rabbit, sheep, dog, cow, and pig.

Patellar tendinitis in athletes is a chronic injury caused by repeated pulling on the patellar tip attachment of the patellar tendon due to excessive training, which can overload the patella and patellar tendon. To study the pathogenesis and development of patellar tendon fibrosis, a rabbit model of patellar tendon fibrosis was established by electrical stimulation-induced jumping (Liu et al., 2020). Intense exercise can cause acute injury to the proximal patella. Wang et al. detected the effect of the time of training after injury on healing (Wang et al., 2017). Xu et al. established a rabbit model of partial patellar resection to verify whether combined magnetic field technology can promote the healing of the bone–tendon junction through endochondral ossification (Xu et al., 2014). Kim et al. evaluated the healing capacity of injected atelocollagen as a treatment scaffold for patellar tendon defects (Kim et al., 2021). Other studies have compared the effects of human recombinant epidermal growth factor and platelet-rich plasma on the histological healing process and gene expression profile using a rabbit patellar tendon incision model (Lyras et al., 2016; Sarıkaya et al., 2017).

The sheep knee joint is often used as a model in orthopedic research. Kayser et al. provided ultrasound images of the sheep knee joint that were relevant in musculoskeletal research (Kayser et al., 2019). The biomechanical characteristics of the patellar tendon, such as elasticity and stiffness, are of paramount importance and constitute major outcome measures in research. Kayser et al. assessed whether the stiffness of the healthy ovine patellar tendon changed with age and weight in a population of normal animals (Kayser et al., 2022). Thangarajah et al. used a sheep model of acute tendon contraction to study the role of the demineralized bone matrix in the treatment of tendon tear retractions. The patellar tendon was detached from the tibial tuberosity and a complete distal tendon transverse defect measuring 1 cm was created. The tendon was reconnected with suture anchors, and the defect was bridged with a demineralized bone matrix and minimally invasive mesenchymal stem cells (Thangarajah et al., 2016). Enea et al. performed surgery on the right hind leg of 48 Welsh goats, removed the central third of the patellar tendon, replaced it with an implant, and studied the effect of implanted collagen on tendon and ligament tissue regeneration *in vivo* (Enea et al., 2013).

One study examined the biomechanical and histological properties of the medial third of the patellar tendon in dogs; the results found a direct contrast to those of the central third (Linder et al., 1994). Gersoff et al. used the canine patellar tendon defect model to perform full-thickness proximal and distal flap defects of the patellar tendon in eight purpose-bred research mongrel dogs, and compared the healing of the Artelon patch-augmented tendon and tendon repair alone (Gersoff et al., 2019). A total of 27 patellar tendons in male Beagles were surgically subjected to stretching by a small diameter or a large diameter rod to induce damage due to strain, and were evaluated at 4- and 8-week intervals using quantitative magnetic resonance imaging, biomechanical testing, and histology (Pownder et al., 2021). de Moya and Kim quantified changes in the patellar tendon length following surgical correction of medial patellar luxation in dogs and evaluated the potential risk factors associated with patellar tendon elongation using dogs that underwent surgery for medial patellar luxation correction and had 2–3 months follow ups (de Moya and Kim, 2020).

In animal models of tendinopathy, the main *ex vivo* measurements of interest are the mechanical properties of the tendon. Reduced mechanical properties leading to an increased likelihood of spontaneous rupture is the result of clinical tendinopathy. The bovine patellar tendon is often used in biomechanical studies owing to its large size. Flanigan et al. evaluated the biomechanical properties of FiberWire, a novel suture material, compared with standard Ethibond sutures for tendon injury repair in the bovine knee joint (Flanigan et al., 2011). A three-step tensile stress-relaxation test was conducted on the patellar tendons of bovines, and the result revealed that long-term relaxation behavior is affected or implied by proteoglycans and crimp angle, possibly relating to slow structural reorganization of the tissue (Ristaniemi et al., 2021). To describe and compare the characteristics and coordination between knee ligaments and patellar tendons, dumbbell-shaped tensile specimens were cut from bovine knee ligament and patellar tendon for tensile testing. This study improved the understanding of the elasticity, viscoelasticity and failure characteristics of knee ligaments and patellar tendons (Ristaniemi et al., 2018).

Neovascular embolization is a therapeutic strategy for chronic musculoskeletal pain. Ghelfi et al. established a large animal model of patellar tendinopathy with neovascularization by percutaneous injection of increased doses of collagenase in nine 3-month-old male piglets. The model is feasible, safe and reproducible, which is helpful for the study of a new treatment for direct endovascular embolization of neovascularization (Ghelfi et al., 2021).

### Achilles tendon injury model

As the largest and longest tendon of the human body, the Achilles tendon can typically bear more than 12.5 times the weight of the individual. Long-term, high-intensity load increases the incidence of Achilles tendinopathy (Komi, 1990). The Achilles tendon is also one of the most thoroughly researched elements of animal models of tendinopathy. It is advantageous to study because of its superficial parts, convenience of operation, and ease of sampling, which are conducive to the study of the mechanisms of tendinopathy. The Achilles tendon has been widely used in a variety of animal models (Hast et al., 2014; Loiselle et al., 2016). Large animals such as sheep or cattle are popular due to the appropriate size of their Achilles tendon, the weight bearing similar to humans, and their suitability for clinical evaluation. Rabbits are also suitable for Achilles tendon injury models because their Achilles tendon size allows for surgical approaches and accurate specimen examination (Doherty et al., 2006).

Skalec et al. conducted an anatomical and histological analysis on eight female New Zealand rabbits and comprehensively described the macroscopic and microscopic morphology of their Achilles tendon and its related structures (Skalec et al., 2019). The Achilles tendon transection model is a common model of injury that is used to study the biomechanical properties of healed tendons and the degree of adhesion formation (Meier Bürgisser et al., 2016), as well as the time-dependent changes of strain ratios (SRs) and the correlation between SRs and mechanical and histological properties of healed tissue (Yamamoto et al., 2017). It was also used to compare the effects of early activity and fixation on postoperative healing of rabbit Achilles tendon rupture (Jielile et al., 2016). The healing of tendons through open and percutaneous repair techniques was compared by histological, electron microscopic and biomechanical investigation (Yılmaz et al., 2014). Achilles tendon defects occur frequently in traumatic injuries. The rabbit Achilles tendon defect model was used to evaluate the repair effect of decellularized bovine tendon sheets (Zhang et al., 2018), polyethylene terephthalate artificial ligaments (Li et al., 2016), and collagen implants with or without a polydioxanone sheath for Achilles tendon defect reconstruction (Meimandi-Parizi et al., 2013). The Achilles tendinopathy model induced by bilateral Achilles tendon injection of collagenase in rabbits accurately represents the progressive histological and biomechanical characteristics of human chronic Achilles tendinopathy (de Cesar Netto et al., 2018). A rabbit model of ischemic injury caused by Achilles tendon ligation was used to compare a series of changes in Achilles tendon morphology and strain in the early stage of Achilles tendinopathy (Ahn et al., 2017).

Achilles tendon rupture is common in sheep Achilles tendon injury models. Previous studies have evaluated the repair effect of cross-linked acellular porcine dermal patches, platelet-rich plasma fibrin matrixes (Sarrafian et al., 2010), exogenous growth differentiation factor CDMP-2 (Virchenko et al., 2008) and plasma rich in growth factors (Fernández-Sarmiento et al., 2013) on sheep Achilles tendon ruptures. Bruns et al. studied the spontaneous healing process of sheep Achilles tendons after transection and partial resection by means of histological and biomechanical analyses (Bruns et al., 2000). Dündar et al. used the sheep Achilles tendon tear model to compare the biomechanical properties of modified Kessler, Bunnell and Tsuge technology in repairing sheep Achilles tendon tears (Dündar et al., 2020). Leung et al. simulated bone–bone, bone–tendon and tendon–tendon repairs with osteotomy of the calcaneus, reattachment of Achilles tendon to the calcaneus after removal of the insertion, and tenotomy of the Achilles tendon resection in 47 goats (Leung et al., 2015). There have also been studies using collagenase injections to create Achilles tendon injury models. Serrani et al. used real-time elastosonography to monitor the progress of Achilles tendon healing after an experimentally induced tendinopathy (Serrani et al., 2021). Facon-Poroszewska et al. compared the efficacy of radial pressure wave therapy with injections of autologous adipose-derived stem cells or platelet rich plasma in the therapeutic procedure for collagenase-induced Achilles tendinopathy in sheep (Facon-Poroszewska et al., 2019).

Cattle Achilles tendons are larger and are often used for improvements in surgical suture techniques. Tian et al. designed the Locking Block Modified Krackow (LBMK) peri-tendon fixation technique for minimally invasive surgery and then compared the biomechanics of LBMK with Kessler and percutaneous Achilles repair system techniques with a simulated early rehabilitation program (Tian et al., 2020b).Tian et al. used 20 fresh bovine Achilles tendon specimens and randomly divided them into two groups, which were respectively sutured by open Giftbox Achilles tendon repair and minimally invasive LBMK techniques. The early rehabilitation simulation scheme was used to compare the biomechanics of the two techniques (Tian et al., 2020a).

There are few studies on pigs as animal models for Achilles tendon injury. Previous studies used pigs to study the biological characteristics of Achilles tendons or collected the Achilles tendons of pigs as materials for tendon injury repair. Zhang et al. characterized the structural components, vascularity, and resident tendon cells of the porcine Achilles tendon (Zhang et al., 2021). Lohan et al. achieved tendon-like tissue formation by implanting decellularized porcine Achilles tendons that were recellularized with human hamstring tendon-derived tenocytes into nude mice (Lohan et al., 2018).

### Flexor tendon injury model

Animal models involving flexor tendons include the superficial flexor digitorum and flexor digitorum profunda tendons. The tendon healing process here is very slow due to the presence of ischemic and cell deficiency, which is consistent with the healing characteristics of tendinopathy (Adams and Habbu, 2015). Flexor tendons are relatively small, limiting their use in small animals. At the same time, large animals have the advantage of naturally occurring tendinopathy (Longo et al., 2011). At present, large mammals such as sheep and horses are the main animal models of flexor tendon injuries. However, large animals cost more, and rabbits offer a good compromise. Moreover, rabbit flexor tendons are more like human tendons in diameter and the presence of a perceptible synovial sheath (Bottagisio and Lovati, 2017).

To understand the repair process of flexor tendon injuries, studies have used the rabbit flexor tendon injury model to detect the expression of mast cells, fibroblasts, neuropeptides (Berglund et al., 2010) and growth response factor-1 (Derby et al., 2012). Progressive tendon adhesion is a major challenge in flexor tendon repair. Liao et al. developed an anti-adhesion scaffold for surgical repair of the flexor tendon in a rabbit model (Liao et al., 2018). Chen et al. investigated the preventive effects and mechanism of chitosan on tendon repair adhesion in rabbit flexor tendons (Chen et al., 2015). Previous studies have explored the effects on the healing of flexor tendon injury in rabbits investigated fibrin glue (He et al., 2013), autologous platelet-rich fibrin (Liao et al., 2017), bone marrow mesenchymal stem cells (He et al., 2015), adipose-derived stem cells (de Lima Santos et al., 2019), growth differentiation factor-5 (Henn et al., 2010), and lactoferrin peptide (Håkansson et al., 2012). A reinforced tubular, medicated electrospun construct was developed for rabbit deep flexor tendon repair that combines mechanical strength with the release of anti-inflammatory and anti-adhesion drugs (Peeters et al., 2022).

Sheep flexor tendon injury models include flexor tendon transection, collagenase induction, and partial tendon resection to create defects. To explore the mechanism of flexor tendon injury, Biasutti et al. recorded the gene expression, and histopathology and biomechanical changes that occur throughout the superficial digital flexor tendon up to 16 weeks post-injury in a sheep surgical model (Biasutti et al., 2017). Previous studies have investigated the repair effects of synovial multipotent cells (Khan et al., 2020), peripheral blood-derived mesenchymal stromal cells, and platelet-rich plasma (Martinello et al., 2013) on experimentally injured deep digital flexor tendons of sheep. The effects of the multiwave locked system were investigated in the acute phase of collagenase-induced tendon lesions in six adult sheep (Iacopetti et al., 2015). De Mattos et al. examined the effect of nano light emitting diode phototherapy on tendinopathy by partial tenotomies measuring 0.2 × 0.5 cm on the second third of the superficial flexor tendons of 10 healthy sheep (de Mattos et al., 2015). The sheep flexor tendon injury model is also used to improve surgical suture techniques. Uslu et al. randomly divided 60 fresh sheep forelimb flexor digitorum profundus tendons into three groups, and used two-, four-, and eight-strand suture techniques, respectively, to investigate the biomechanical relationship between the diameter of the core suture and the final repair strength of the surrounding suture with an increase of the number of suture lines (Uslu et al., 2014). Doğramaci et al. used 20 fresh flexor digitorum profundus tendons from the forelimbs of healthy adult sheep to improve suture techniques and evaluate their mechanical properties after repair (Doğramaci et al., 2009).

The flexor digitorum superficialis tendon of horses is a frequently injured structure that is functionally equivalent to the human Achilles tendon. Both play a key role in energy storage systems during high-speed exercise and can accumulate microdamage related to exercise and age, and are prone to rupture during strenuous activities (Patterson-Kane et al., 2012). To study the biological mechanism of age-related tendon injury, some studies have performed qualitative and quantitative analyses on the gene expression and collagen fiber diameters of the flexor digitorum superficially and the flexor digitorum profunda tendons of horses at different ages (Ribitsch et al., 2020). Some researchers studied the therapeutic effect of fetal-derived embryonic-like stem cells using a collagenase gel-physical defect model in the mid-metacarpal region of the superficial digital flexor tendon of horses (Watts et al., 2011). Durgam et al. described the value of intralesional tendon-derived progenitor cell injections in equine flexor tendinitis using collagenase-induced tendinitis in both front superficial digital flexor tendons of horses (Durgam et al., 2016). Some studies examined superficial flexor tendon injuries in both horse forelimbs to explore the safety and effectiveness of equine allogeneic tenogenic primed mesenchymal stem cells in the treatment of tendon injury (Depuydt et al., 2021), and compared the changes of imaging, histology, and biochemical and biomechanical parameters (Johnson et al., 2021). Nelson et al. compared the intra- and postoperative clinical features of desmotomy of the accessory ligament of the superficial digital flexor tendon using a Saber radiofrequency electrosurgical probe *versus* sharp transection with a tenotomy knife (Nelson et al., 2015). With in-depth research on equine tendinopathy in recent years, the application of flexor tendon modeling is more common, and further promotes the translation of research results from large animal models to clinical practice on humans.

## Conclusion

The selection of preclinical models is essential to ensure that the efficacy and safety of studied treatments is successfully translated into clinical practice. The selection of animal models requires consideration of scientific criteria, economics, and ethical issues. Indeed, the costs associated with animal rearing and management significantly influence the selection of animal species because an adequate sample size is necessary to obtain reliable results. Therefore, the selection of animal models for proof-of-concept studies needs to balance the ethical justification, cost-effectiveness, and appropriateness of the model itself, such as anatomical location, size, surgical approach, and biomechanical properties. When considering the efficacy of a new therapy, for the validity of preclinical models, the identification of appropriate controls, optimal study duration and intermediate time points, and the provision of the most in-depth analysis must be considered (Bottagisio and Lovati, 2017).

Rodents are the most used animal model for the study of the genetic and molecular mechanisms of tendinopathy due to their minimal cost and ethical compliance. Rats are often used to explore the factors affecting tendon-bone healing (Degen et al., 2016; Arimura et al., 2017; Nakagawa et al., 2017; Yonemitsu et al., 2019), the molecular mechanisms underlying ectopic ossification in tendinopathy (Geng et al., 2020) and inflammation and scar formation in the injury tendon healing (Wang et al., 2019; Geng et al., 2020). The advantage of mouse model is the ability to examine the role of specific signal transduction pathways and molecules in tendon degeneration and repair by gene knockout (Kuenzler et al., 2017; Jensen et al., 2018). Despite their low cost and ease of rearing, the small size of rats and mice has an impact on surgical approaches, non-invasive imaging techniques, and biomechanical testing. Moreover, the relevant results still need to be applied to large animals and clinical trials to ensure safety and effectiveness.

Compared with small animals, large animals share many similar characteristics with humans in genetics, anatomy, physiology, pathology, and so on. Therefore, large animals are more suitable for macroscopic research on topics such as tendon biomechanics, scaffold repair technology, and surgical method optimization. The advantages and disadvantages of large animal models in tendinopathy research are summarized in Table 1. However, due to the prohibitive cost of rearing and managing large animals and the difficulty of ethical approval, typically only small sample studies can be conducted. Rabbits offer a satisfactory compromise. The size and biomechanical properties of rabbit tendons are similar to those of human flexor tendons (Goodman and Choueka, 2005; Nagasawa et al., 2008), allowing for surgical and *in vivo* imaging analysis. The docility of rabbits allows evaluation of postoperative rehabilitation effects by immobilization or active loading. Rabbit models can also be used to evaluate the role of autologous cell therapy and regenerative medicine (Chong et al., 2007). Sheep are readily available, moderately expensive, and are used to study chronic tendon injuries and various suture techniques to reduce postoperative adverse conditions. Dogs are used to explore the repair effect of tendon injury mediated by internal factors and postoperative rehabilitation schemes, owing to their high degree of cooperation. However, public opinion pressure against the animal testing dogs hinders the selection of this species. Some experiments have used specially bred dogs as models of tendinopathy. The horse is a natural tendon injury model due to its frequent sports. Horses can be used to study the mechanisms and repair methods of tendon injury. Porcine tendons and ligaments are the best animal sources for xenogeneic tendon transplantation.

**TABLE 1:** Advantages and disadvantages of large animal models in studying tendinopathy.

Species	Advantages	Disadvantages
Rabbit	Low cost and easy to access and manage	Strong self-healing ability, not easy to simulate the disease process
	Cytological and histological levels similarities to humans	Diarrhea occurs easily due to fright Lui et al. (2011)
	Medium somatotype is convenient for surgical operation	
	Biomechanical testing can be performed	
	*In vivo* imaging can be performed	
	Commercial reagents are available for molecular research	
	The rabbit subscapularis muscle model, which has a similar structure with humans, can be used for the study of rotator cuff injury Grumet et al. (2009)	
Sheep	Easily available and feeding	High costs of feeding and management
	Biomechanical and anatomical similarities to humans	Long growth period
	Big dimensions and easy to perform surgical procedures	Not useful for rehabilitation programs
	Biomechanical testing can be performed	
	Model transformation can be carried out	
	*In vivo* imaging can be performed	
	It is mainly used in the study of chronic tendon injuries and various suture techniques to reduce postoperative adverse conditions Gerber et al. (2004)	
Canine	Easy to access and manage	Higher costs of purchase and breeding
	Similar biomechanical environments	Difficulty unfolding large sample experimental studies
	Large dimensions and easy to perform surgical procedures	Significant differences from the anatomy of humans
	Biomechanical testing can be performed	Cases with spontaneous rotator cuff degeneration, not conducive to control variables Fransson et al. (2005)
	Model transformation can be carried out	Ethical concerns
	Better tolerance of multiple postoperative rehabilitation programs	
	*In vivo* imaging can be performed	
	It is used to explore the repair effect of tendon injury mediated by some internal factors and postoperative rehabilitation plan	
Cattle	Offer great potential for long-term functional studies	High feeding costs
	It is used in biomechanical research	Lack of reagents for molecular studies
Horse	The flexor digitorum superficialis tendon of horse is functionally equivalent to the human Achilles tendon	High feeding costs
	Tendinopathy occurs naturally Longo et al. (2011)	Lack of reagents for molecular studies
Primate	The anatomical structure and physiological functions are closest to those of humans	The cost of purchasing, feeding and management is extremely high
	Larger tissues allow for easier examination	Ethical concerns
	The structure of the shoulder is very similar to that of humans. Baboons may be the best animals to study rotator cuff damage Sonnabend et al. (2010)	

The application of animal models has promoted the progress of tendinopathy research but, given the complexity of human tendinopathy, there are still significant differences between animal experimental models and clinical human tendon injuries. In addition, more validated animal models are needed, as no single model can answer all the questions (Warden, 2007). It is hoped that, with the further development of animal models of tendinopathy, new strategies for the prevention and treatment of human tendinopathy can be provided.
